# Hearing abilities stimulation program for schools

**DOI:** 10.1590/2317-1782/e20240065en

**Published:** 2025-03-31

**Authors:** Ana Flávia de Oliveira Nalom Baruchi, Eliane Schochat

**Affiliations:** 1 Departamento de Fonoaudiologia, Fisioterapia e Terapia Ocupacional, Faculdade de Medicina, Universidade de São Paulo – USP - São Paulo (SP), Brasil.

**Keywords:** Hearing, Auditory Perception, Acoustic Stimulation, Reading, Learning

## Abstract

**Purpose:**

To develop and verify the effectiveness of a hearing abilities (HA) stimulation program included in the regular school curriculum and applied by teachers in the classroom.

**Methods:**

An HA stimulation program was developed and applied to preschoolers during the school year; 34 children underwent auditory stimulation (ASG) and were compared to a placebo group (PG; n = 31). The students were assessed regarding their HA and pre-reading and decoding skills before and after the intervention. They were reassessed twice after applying the program to monitor the students' performance amid the COVID-19 pandemic.

**Results:**

The program includes activities applied at school by teachers for 25 weeks, lasting 10-15 minutes/day, stimulating the following HA: detection, discrimination, temporal processing, figure-ground, closure, memory, and attention. The ASG and PG performances differed significantly after the program.

**Conclusion:**

The program was incorporated into the curriculum, helping to develop the skills recommended by the Ministry of Education. After auditory stimulation, ASG performed better in auditory figure-ground, temporal resolution, rhyme identification and production, and word production from the phoneme given.

## INTRODUCTION

Important auditory and linguistic acquisitions mark the first years of a child's life. During this period, the central nervous system is constantly being molded and modified in what we briefly know as neural plasticity. Their auditory experiences at this stage are fundamental for satisfactory development, as the environment (and its stimuli) modulate and increase the auditory nerve activity, providing the child with satisfactory speech perception over the years^(1)^.

According to Jain et al.^(2)^, temporal processing skills are crucial in the first 7 years of a child's life and play an important role in the knowledge of speech sounds (discrimination of similar vowels and other linguistic sounds) and in language acquisition/development. In this sense, such skills are related to the ability to identify and manipulate speech segments – i.e., phonological awareness.

A longitudinal study by Vanvooren et al.^(3)^ followed 87 five-year-old children at high and low family risk for dyslexia as they learned to read and write. The authors found a positive relationship between preschool hearing abilities (HA) and subsequent phonology and literacy performance. In other words, while the processing of temporal speech signals is linked to phonological skills, auditory closure was a predictor of literacy.

Other studies^(4,5)^ indicate an association between temporal auditory processing (AP) and oral and/or written language disorders. According to these authors, such hearing difficulties may occur within the speech spectrum and interfere with the formation of phonological representations, which would be reflected in graphophonemic association. Thus, the adequate functioning of the auditory system is a prerequisite for an effective teaching-learning process^(6)^.

Stimulation of the auditory nervous system can improve the efficiency of the auditory system. Auditory stimulation activities aim to strengthen the central auditory nervous system and related systems, modifying the child's behavior in response to content that reaches them through the auditory pathway in daily tasks (including academic ones)^(7)^.

The Brazilian National Curriculum Framework (BNCC, in Portuguese) recommends that preschoolers be encouraged and enabled to identify sounds produced by different objects, recognizing their qualities (intensity, duration, pitch, and timbre) to prepare them for the next stage^(8)^.

This justifies the relevance of this research, differing it from other studies by incorporating auditory activities into the regular curriculum to help students acquire the alphabetic writing system in articulation with HA development (as recommended by the BNCC), at a low cost to educational institutions, using materials available in educational units. Hence, this study aimed to develop and verify the effectiveness of a program with recreational activities to stimuli HA, inserted into the students’ regular curriculum in the last year of preschool and carried out by teachers in the classroom, and verify its impact on the stimulated children’s pre-reading skills.

## METHOD

This longitudinal, prospective, analytical, interventional study was approved by the Research Ethics Committee of the Medical School of the University of São Paulo (protocol no. 3.469.029).

The person responsible for the participating institution signed an Educational Unit Authorization Form. The participating students signed an assent form, and their teachers and parents signed an informed consent form.

The study data were collected at a regular public municipal school in an inland city in São Paulo, Brazil, and were stored at the Speech-Language-Hearing Neuroaudiology Research Laboratory of the Speech-Language-Hearing program at the Medical School of the University of São Paulo.

The room chosen for the assessments had the lowest sound pressure level (SPL) in the school, close to the level permitted for environmental noise in institutions according to the Brazilian Association of Technical Standards (ABNT). An Instrutherm SPL meter, model DEC-460, measured the average environmental noise level, following its instructions for use, and finding it was 65 dB. The researchers also considered the pedagogical demand and the lack of flow of people in these spaces.

The inclusion criteria for this study were being regularly enrolled in the last year of preschool at the selected school and having no indicators of hearing changes (history of recurrent otitis or family hearing loss) or sight changes and no family history or signs and symptoms of neurological, behavioral, or cognitive changes (screened through questionnaires).

The study included 65 children enrolled in the last year of preschool at the selected school, who had 75% or more attendance in classes during the school year in which the activities were carried out. They were divided into two groups:

- **Placebo group (PG):** 31 students who received placebo stimulation of visual and psychomotor skills for 10-15 minutes daily, for 25 weeks of the 2019 school year.
- **Auditory stimulation group (ASG):** 34 students who received auditory stimulation for 10-15 minutes daily, for 25 weeks of the 2019 school year.

The children belonged to four classes at the same school. Two classes were randomly selected to form the PG and two classes to form the ASG.

The study had three stages: initial assessment; development and application of the HA stimulation program; and reassessment.

### Initial and final assessments

Two assessments were carried out: M1 (initial assessment) and M2 (reassessment after applying the program). Tests were applied to assess HA and pre-reading and reading skills, as described in the test manuals.

Initially, it was planned to assess all students’ hearing (more information under study limitations). However, it could not be carried out due to unforeseen events during the research – it was affected by the COVID-19 pandemic, making it impossible to constantly transport the equipment from the laboratory and the partner school, which are in different cities. Since the HA assessment was not meant for diagnosis but to help observe the children’s performance before and after HA stimulation, behavioral tests were applied, maintaining the same level of stimulus presentation for all subjects (described below). The results were compared between groups and between subjects – hence, without test normality, used for formal soundproof booth assessment after identifying individual hearing thresholds. This was an option of the authors, considering that the age range evaluated was below the normality of the tests, hindering comparisons between expected values ​​at different ages.

In this process, one child had difficulty performing the tests at the established level and was referred for a formal hearing assessment, which identified mild sensorineural hearing loss in the right ear (RE) and moderate sensorineural hearing loss in the left ear (LE). This child participated in the program with the other students but was not included in the ASG assessments.

All children’s external auditory meatus were inspected before all AP screening procedures, which assessed behavioral responses to the following tests:

1. Dichotic Digits Test^(9)^ (DDT) for binaural integration. They had to identify and repeat four different numbers presented simultaneously to both ears.
2. Masking Level Difference^(10)^ (MLD) for binaural interaction, in binaural conditions, presenting 33 segments of narrowband noise in one ear, for at least 3 seconds, with or without a 500 Hz pure-tone stimulus. Three conditions were considered: pure-tone and narrowband phase noise in both ears (homophasic signal/noise condition - SoNo); inverted phase pure tone in one ear and phase noise in both ears (signal/noise condition SπNo); and noise without pure tone (no tone - NT). The children were instructed to raise their hands every time they heard the whistle (pure tone). The times when participants indicated hearing a sound were summed for analysis. Then, this number was converted to dB, following the table in the test.
3. Random Gap Detection Test^(11)^ (RGDT – standard version) for temporal resolution. Pure tones were presented to the children at 500, 1000, 2000, and 4000 Hz, binaurally. The paired pure tones were presented randomly at time intervals of 0 to 40 ms, with increments ranging from 2 to 10 ms. At each presentation, the children were instructed to point at an image containing one or two squares (■ or ■■) or say whether they were hearing one or two sounds.

All tests were applied using a notebook and inspected headphones (Sony wired MDR-7506) and were previously trained. A Svantek^®^ SV-102^®^ noise dosimeter evaluated the stimulus presentation level, with a microphone plugged into the outputs of the headphones, and the computer at its medium volume. The equipment captured the following mean SPL: DDT – 58 dB; MLD – 58.1 dB; RGDT – 500 Hz: 54.1 dB | 1000 Hz: 54.1 dB | 2000 Hz: 57.7 dB | 4000 Hz: 51.4 dB.

Two test application sequences were used (DDT + RGDT + MLD or MLD + RGDT + DDT) to eliminate the “order of application” bias in the results and interpretation of the AP screening tests. Each sequence was used with 50% of the subjects in each group.

The hearing tests were applied in 40 minutes on average, considering the need for explanations appropriate for the students’ age and verbal comprehension level.

The Protocol for Early Identification of Reading Problems, proposed by Capellini et al.^(12)^, was chosen to assess pre-reading and reading skills. Not all items of the protocol were applied since the preschoolers had not yet acquired all skills assessed by the instrument. However, an attempt was made to apply examples of all skills to identify in which of the assessments a new skill could be observed. The average time for applying this protocol was 25 minutes.

The skills assessed in this research were:

- Letter identification by presenting them to students for them to identify the letter name and sound.
- Rhyme production through the auditory presentation of 20 words for them to say a word that ended with the same sound.
- Rhyme identification through the auditory presentation of 20 trios of words to identify those ending with the same sound.
- Syllabic segmentation through the auditory presentation of 21 words to be divided into syllables.
- Word production from the phonemes of the alphabet.
- Phonological working memory from the repetition of 24 non-words.
- Fast automatic naming of seven interleaved sequences of colored drawings (car, ball, duck, house, and key).
- Visual attention from a silent reading of word pairs for association with a target figure.
- Reading aloud 20 words and 20 non-words.
- Comprehension of 20 incomplete sentences with associative illustrative figures presented for students to orally complete the sentences.

The reading skills were assessed on a different day to that of the HA, in the same room where the AP assessment tests were applied.

### Developing the auditory abilities stimulation program

The program activities were developed considering the following HA: detection, discrimination, figure-ground, closure, temporal processing (discrimination of frequency, duration, and intensity patterns), memory, and attention. The tasks also stimulated sound localization indirectly throughout the year.

The aim was to respect the natural order of HA acquisition and development, the comprehension level expected for their age, materials easily accessible to the participating public school (previously consulted by the researcher at the school), and the teachers' understanding of the activities to be implemented and the importance of their objectives. It also considered the different classroom organizations and configurations. To minimize this impact and allow students to receive the target stimuli from different locations, teachers were instructed to keep the classroom windows and doors closed during the program application. They should also stand in different locations or have the students sit.

When the teachers identified that the children did not understand the task, the school vice-principal immediately contacted the researcher, who changed the instructions or adapted the activity. The activity was considered adequate when both teachers applying it indicated an overall score between 8 and 10 in the evaluation on the back of the weekly explanation given to them.

The program's activities were based on adapted auditory training strategies used in clinical speech-language-hearing practice and research carried out in schools^(13,14)^.

The adaptation suggested for this program aimed at group participation in activities; having some children use other sensory pathways for support (such as the visual pathway); the benefit of learning from auditory stimuli and peer imitation, even using the visual pathway; the differences in each classroom’s physical environment; the precocity at which the stimuli would be presented at school; the teachers’ benefit of learning a new auditory ability per week and observing it in practice; the understanding that materials may vary, as long as strategies influence the functioning of the central auditory nervous system.

The daily auditory activities lasted 10 to 15 minutes. PG children would perform visual or psychomotor activities daily for 10-15 minutes as well.

The researcher verbally guided and trained four teachers (two applying the auditory activities and two applying the placebo activities) on both stimulations every two weeks to ensure a double-blind study. If they had any questions, they should ask the vice-principal to contact the researcher by telephone. The vice-principal mediated between the teachers and the researcher.

Although the objective of this study was not to evaluate each subject’s daily progress, the teachers were invited to evaluate the class in every week’s activity. The weekly dialogue with the school team led activities to be designed according to the challenges encountered, despite the schedule of abilities to be stimulated.

A statistician performed descriptive analyses of the behavioral test results by constructing tables with the descriptive statistical values (mean, standard deviation, by group, and by ear).

The multivariate analysis of variance (MANOVA) and multivariate analysis of variance with repeated measures (repeated measures MANOVA) respectively compared the means of the study variables in the two groups (ASG and PG) in the study periods. MANOVA analyzed the p-value and the F ratio (which tests the overall difference between groups), using the Wilks' Lambda test (Wilks' λ)^(15)^.

The descriptive analysis was complemented with a 95% confidence interval and a 0.05 (p) significance level (5%).

## RESULTS

### Auditory abilities stimulation program

The final version of the program has activities covering 25 weeks of the school year to stimulate detection, discrimination, auditory figure-ground, closure, temporal processing (discrimination of frequency, duration, and intensity patterns), memory, and attention.

The average time taken to implement the activities ranged from 10 to 15 minutes/day. All activities were implemented between 80 and 100% of the suggested days.

Chart 1 shows a final list with all resources used and a summary of the program activities, according to each week’s objective or target skill.

**Chart 1:** **.** List of resources and summary of activities according to the program’s final objectives

**Week**	**Target ability**	**Resources**	**Summary of activities**
**1**	Auditory detection	- Bell, *reco-reco*, shaker, drum, triangle, clapping, and the teacher’s voice	- Presenting the instruments.
			- Identifying sounds in quiet and raising the hand when hearing them.
**2**			Presenting 20 stimuli.
**3**	Auditory memory	- Drum, shaker, *reco-reco*, bell, and coconut shells	- Identifying isolated instruments.
			- Memorizing and identifying 14 instrumental sound sequences with one, two, three, or four stimuli.
**4**	Attention	- Sound system.	- Clapping when hearing the target word of each song played.
		- "Lottie Dottie Chicken” YouTube channel.	- Two songs a day.
		- Selected songs.	
**5**	Discrimination	- Poster with identical and different figures.	- Concept of same and different.
		- Sleigh bells, coconut shells, shaker, and triangle.	- Identifying whether the sounds played were the same or different, using visual support.
**6**		- The teacher’s voice and a word list.	- Presenting 10 to 15 sequences.
**7**	Memory	- Figures of the semantic fields chosen by the teacher (if necessary).	- Playing “I packed my bag” children’s game.
			- 10 minutes.
**8**	Attention	- “*Estrelinha I*” collection (Sônia Junqueira, 2019).	- Explaining the activity.
			- Raising the hand every time the target word of a story from the “*Estrelinha I*” Collection was read.
			- Conversation circle and story comprehension until completing 15 minutes.
**9**	Perception of sound duration pattern	- Musical instruments selected by the teacher.	- Presenting the day’s instrument.
		- Poster with visual aid for the concept of long and short.	- Identifying the difference between short and long sounds.
		- List with the sequence of stimuli.	- Naming the 10 sequences presented.
**10**	Perception of sound frequency pattern	- Drum, triangle, bell, and coconut shells.	- Presenting the day’s instrument.
		- List with the sequence of stimuli.	- Developing the concept of high and low sounds and associate them with the day’s instruments.
			- Naming the 10 sequences presented.
**11**	Memory	- Target-word list.	- Memorizing sequences of words and recalling them, from time to time, during the class.
		- Clock.	- 40 to 60-minute intervals between recalls.
**12**	Attention	- Sound system.	- Explaining the activity.
		- “*Palavra Cantada*” YouTube channel.	- Clapping when hearing the target-word.
		- Selected songs.	- Two songs a day.
**13**	Figure-ground	- Sound system.	- Listening to the story told by the teacher (with 110 to 140 words) while a background musical noise is presented simultaneously.
		- “*Palavra Cantada*” YouTube channel.	- Conversation circle and reading comprehension until completing 15 minutes.
		- Selected songs.	
**14**		- A story selected by the teacher.	
**15**	Memory	- Target-word list.	- Memorizing sequences of words and recalling them, from time to time, during the class.
		- Clock.	- 40 to 60-minute intervals between recalls.
**16**	Attention	- Texts selected by the teacher.	- Listening to the story told by the teacher (with 110 to 140 words) and raising the hand when hearing the target-word.
			- Conversation circle and reading comprehension until completing 15 minutes.
**17**	Closure	- Songs selected by the teacher.	- Ignoring the music being played for 15 minutes of routine school activities.
**18**		- Sound system.	
**19**	Memory	- Figures of the semantic fields chosen by the teacher (if necessary).	- Playing “I packed my bag” for 15 minutes.
**20**	Attention	- Utensils selected by the teacher.	- Presenting the day’s four objects to the students.
			- Identifying the objects’ sounds.
			- Naming 15 sequences with three sounds each played by the teacher.
			- Identifying the missing sound in the sequence played.
**21**	Detection	- List of linguistic stimuli.	- Raising the hand and keeping it raised while listening to 20 sequences with target syllabic or phonemic stimuli.
		- The teacher’s voice.	
**22**	Discrimination	- Figures selected by the teacher to reinforce the concept of same and different.	- Discriminating words or minimal pairs.
		- Word list.	- 10 sequences a day.
		- The teacher’s voice.	
**23**	Perception of sound intensity pattern	- Shaker, bell, sleigh bells, coconut shells, and drum.	- Presenting the day’s instruments.
		- Stimulus presentation list.	- Identify the difference between high and low-intensity sounds.
			- Naming the 10 sequences presented.
**24**	Figure-ground	- Competing sound selected by the teacher (unknown song, instrumental song, poorly tuned radio).	- Playing “I packed my bag” with background noise.
			- 10 minutes of activity.
**25**	Closure	- List of graphemes or figures prepared by the teacher.	- Dictating 15-20 graphemes a day with competing noise.
		- Guidance to adapt activities.	
		- Songs selected by the teacher.	

### Effectiveness of the application of the HA stimulation program in preschool

The participants’ mean age was 60 months in M1 and 68 months in M2. ANOVA detected no statistically significant difference between the mean ages at any of the evaluated moments nor any difference between the groups: M1 [F(1.63) = 0.24, p = 0.63] and M2 [F(1.63) = 0.24, p = 0.63].

The chi-square test compared the participants’ sex and found no association between group and sex: X^2^ (1) = 0.42 (p = 0.52).

Table 1 presents the mean values ​​and standard deviations of ASG and PG performance in AP tests (DDT, MLD, and RGDT) in M1 and M2.

**Table 1:** Group performance in the auditory processing tests before and after the intervention (M1 and M2)

			**ASG**				**PG**			
			M1		M2		M1		M2	
			**Mean**	**Standard deviation**	**Mean**	**Standard deviation**	**Mean**	**Standard deviation**	**Mean**	**Standard deviation**
DDT	RE		53.53	13.07	72.79	15.53	53.55	13.05	60.48	12.67
	LE		52.13	16.82	72.06	16.77	50.76	13.66	59.71	14.86
MLD			7.88	3.75	6.53	4.22	9.19	5.63	6.90	4.06
RGDT			11.14	4.40	9.74	3.61	14.65	6.90	13.95	4.94

**Caption:** ASG – auditory stimulation group; PG – placebo group; RE – right ear, LE – left ear; M1 – moment one; M2 – moment two; DDT – Dichotic Digits Test; MLD – Masking Level Difference; RGDT – Random Gap Detection Test

The repeated measures MANOVA revealed a statistically significant effect between the M1 and M2 performances [F (4.60) = 22.12, p < 0.001***, partial η^2^ = 0.60, Wilks' λ = 0.40]. The univariate analysis revealed a statistically significant difference between M1 and M2 in all central AP tests (p < 0.05).

Furthermore, the analysis revealed a significant interaction between moments (M1 and M2) and interventions (ASG and PG): [F (4.60) = 4.10, p = 0.005**, partial η^2^ = 0.22, Wilks' λ = 0.78].

Statistically significant differences between ASG’s and PG’s performances occurred in DDT RE (p = 0.002**) and DDT LE (p = 0.008**).

The interaction between period and group was further investigated using the t-test. Given that there are two simple effect tests, the significance criterion was set to 0.025.

The analysis found a statistically significant difference in ASG’s performance before and after the intervention for DDT RE [t(33) = -6.76, p < 0.001***], DDT LE [t(33) = -7.31, p < 0.001***] and RGDT [t(33) = -6.76, p < 0.001***]. However, no statistically significant difference was observed in MLD [t(33) = 1.54, p = 0.132].

It also found a statistically significant difference in PG’s performance before and after the intervention for DDT RE [t(30) = -2.80, p = 0.009**] and DDT LE t(30) = -2.99, p < 0.005**]. There was no statistically significant difference in PG’s MLD [t(30) = 2.06, p = 0.04] and RGDT [t(30) = 0.57, p = 0.567] tests.

Table 2 presents the mean values ​​and standard deviations of ASG’s and PG’s performances in the pre-reading and reading skills tests in M1 and M2. This analysis had fewer individuals in the PG because two children did not complete the reassessment.

**Table 2:** Group performance in pre-reading and decoding skills tests before and after the intervention (M1 and M2)

	ASG (n = 34)				PG (n = 29)			
	M1		M2		M1		M2	
	Mean	SD	Mean	SD	Mean	SD	Mean	SD
Number of correct answers in the letter identification test	78.40	21.37	97.06	5.32	65.37	29.30	88.01	24.31
Number of correct answers in the rhyme production test	0.00	0.00	11.32	17.94	0.00	0.00	3.62	7.43
Number of correct answers in the rhyme identification test	0.00	0.00	50.74	31.00	0.00	0.00	20.86	23.98
Number of correct answers in the syllabic segmentation test	18.05	35.04	88.00	13.59	0.00	0.00	62.29	28.25
Number of correct answers in the word production test from a given phoneme	31.10	27.11	69.74	23.13	42.60	30.36	57.60	31.40
Number of correct answers in the phonological working memory test	75.97	13.30	92.46	9.41	73.95	25.25	83.14	18.38
Time, in seconds, in the rapid automatized naming test	57.03	11.09	46.12	10.90	49.76	16.45	44.48	9.67
Number of correct answers in the silent reading test	55.29	19.73	76.18	17.76	56.21	24.56	64.48	21.97
Number of correct answers in the word and pseudoword reading test	0.29	1.72	5.59	16.04	0.00	0.00	4.74	13.03
Number of correct answers in the sentence listening comprehension test	68.53	10.48	83.38	17.00	68.62	23.68	79.48	15.77
Number of correct answers in the phonemic synthesis test	0.00	0.00	0.00	0.00	0.00	0.00	0.00	0.00

**Caption:** ASG – auditory stimulation group; PG – placebo group; n – number; M1 – moment one; M2 – moment two; SD – standard deviation

The repeated measures MANOVA revealed a significant effect between M1 and M2 performances [F (10.52) = 87.79, p < 0.001***, partial η^2^ = 0.94, Wilks' λ = 0.05]. The univariate analysis revealed a statistically significant difference between M1 and M2 in all pre-reading and reading skills tests (p < 0.01) – except for the phonemic synthesis test, whose percentage of correct answers was 0 in both groups for M1 and M2 assessments.

Statistically significant differences between ASG and PG performances occurred in four variables, namely: rhyme production (p = 0.03*), rhyme identification (p < 0.001***), word production (p < 0.001***), and Working Memory (p = 0.04*).

The interaction between period and group was further investigated using the t-test, with the significance criterion adjusted to 0.025.

The analysis found a statistically significant difference in ASG’s performance before and after the intervention for the tests of alphabetic comprehension [t(33) = -5.77, p < 0.001***], rhyme production [t(33) = -3.68, p = 0.001***], rhyme identification [t(33) = -9.55, p < 0.001***], syllabic segmentation [t(33) = -12.15, p < 0.001***], word production [t(33) = -8.71, p < 0.001***], working memory [t(33) = -10.78, p < 0.001***], naming stimuli [t(33) = 7.38, p < 0.001***], silent reading [t(33) = -5.55, p < 0.001***], and auditory comprehension [t(33) = -4.83, p<0.001***]. No statistically significant difference was found in the oral reading test [t(33) = -1.94, p = 0.06].

It also found a statistically significant difference in PG’s performance before and after the intervention for the tests of alphabetic comprehension [t(28) = -4.63, p < 0.001***], rhyme production [t(28) = -2.62, p = 0.01**], rhyme identification [t(28) = -4.68, p < 0.001***], syllabic segmentation [t(28) = -11.87, p < 0.001***], word production [t(28) = -3.23, p = 0.003**], working memory [t(28) = -2.79, p = 0.009**] and auditory comprehension [t(28) = -3.22, p = 0.003**]. However, no statistically significant difference was found in naming stimuli [t(28) = 1.54, p = 0.13], silent reading [t(28) = -1.47, p = 0.15], or oral reading [t(28) = -1.95, p = 0.06].

## DISCUSSION

### HA stimulation program

This study’s main objective was to develop an HA stimulation program and verify its effectiveness. It was applied by teachers at school and incorporated into the regular curriculum, considering the importance of early HA stimulation, strongly associated with subsequent learning to read and write.

The development of this study’s program approached activities that could be applied informally at school, at a low cost for the institutions that would use this material in the future.

The program is considered informal because it uses uncontrolled acoustic stimuli through simple materials teachers present live, guided by a manual.

Although the program’s objective was not to train individuals with AP disorder (APD), it considered the guidelines for the different approaches to APD described by ASHA. Therefore, it was decided to address all HA throughout the year (direct skill remediation), consider compensatory strategies in the process of acquiring and developing the abilities stimulated in each activity, and use a wide range of possible environmental changes in the classroom, where students, teachers, and objects circulate constantly.

According to Masquelier^(14)^, these three methods complement each other to provide bottom-up auditory training, together with the recruitment of higher-order brain functions (i.e., the top-down approach). Changing the stimulus presentation environment can maximize the opportunities to process auditory stimuli effectively, as they approximate common situations where abilities are always recruited.

The final program model was structured to take 25 weeks of the school year, five times a week, with an average of 10-15 minutes of daily activities. This short time aims not to harm the content recommended by the Brazilian Ministry of Education and Culture (MEC) and programmed by the teachers while still being sufficient to positively impact the students’ development. The activities included the HA of detection, discrimination, perception of duration, frequency, and intensity patterns, figure-ground, closure, attention, and memory.

The activities are applicable to students in the last year of preschool. Hence, it is suggested that they be tested and, if necessary, adapted for first graders.

### Effectiveness of the application of the HA stimulation program in preschool

The results generally indicate that both groups performed better as the assessments progressed (as expected for child development), and that the ASG performed better than the PG after auditory stimulation.

The ANOVA did not detect any statistically significant difference between the mean ages at any of the times evaluated. All children in the study were within the ideal age to attend the last year of preschool, according to the MEC.

No associations were found between groups, sex, and age (p = 0.52), demonstrating a balance between boys and girls in both groups.

Both groups had better results in all tests applied in M2 than in M1, which can be justified by the study children’s developmental stage. The first years of a child's life are essential for the central auditory nervous system to continue the development initiated in intrauterine and neonatal life^(16)^. The auditory nervous system has greater plasticity and maturates in the first childhood years, establishing new neural connections^(17,18,19)^.

According to MANOVA, M1 and M2 interacted significantly with the intervention type (ASG had higher results than PG in M2). Thus, it can be stated that presenting playful HA stimulation strategies continuously in the classroom for a short time each day, without the teachers needing to remove any activity from their annual planning, positively favors the skills worked on. This reinforces that children’s positive auditory experiences are essential for the good development and improvement of an efficient auditory perceptual system ^(1,16,20,21)^, directly impacting the development of phonological awareness.

The analysis found statistically significant differences in ASG’s performance between M1 and M2 for auditory figure-ground and temporal resolution. The auditory figure-ground ability for linguistic sounds is essential in communicative environments involving the task of directing attention to the target stimulus competing with other less relevant stimuli. For instance, student in the classroom need to direct attention to the teacher's explanation and understand their message, even though the environment is often noisy.

Similar to the results of the present research, other studies analyzed students’ figure-ground performance after auditory training and reported an evolution of this ability concomitantly with other HA (whether impaired or not)^(22,23)^.

Studies that used noise desensitization training to improve speech perception revealed that this task positively influences the figure-ground ability by simulating real-life demands^(3,21)^.

Similar to the present research, Hassaan and Ibraheem^(13)^ suggested an auditory training program for the figure-ground ability whose material (in Arabic) aimed at desensitization, using a noise presented simultaneously with a story read by the teacher, considering classroom challenges.

After listening to the stories, the subjects were asked questions, demonstrating how much they understood and memorized during the task, expanding the auditory attention spans and memory, critical for learning. Due to the children’s age, the present research replaced formal questionnaires with conversation circles about the text, with the teacher helping reconstruct the story. According to the authors, this form of presenting the stimuli combined informal programs’ flexibility with formal programs’ capacity to modify cortical functioning (although less intensely) since the children's target ability improved.

Chermak and Musiek^(24)^ reported that complex auditory tasks require discrimination of acoustic events, auditory temporal processing, and cognitive actions such as attention and memory. Auditory stimulation aimed at understanding speech in noisy/unfavorable situations can be considered an auditory task complex enough to strengthen the AP globally, explaining why programs to stimulate a specific skill help develop or improve other HA. According to Murphy et al.^(25)^, this improvement is not limited to HA but extends to memory, attention, and language skills, justifying the reading test results described below.

The population of all studies cited above was older than in the present one. Hence, it can be inferred that applying tasks to select a target stimulus competing with others benefited the preschoolers’ HA, as well as their memory, attention, and language skills.

As previously mentioned, the temporal resolution of auditory-stimulated children appears to have benefited from this program’s activities (or from a combined influence of their development process and the plasticity resulting from task learning). Although the stimuli are not precisely controlled (as in formal programs, which use computerized techniques), the activities helped children perceive acoustic variations in time (longer or shorter). According to Musiek et al.^(19)^, temporal resolution is related to perceiving small intervals of silence within or between speech segments. Therefore, speech processing depends partly on temporal processing, which directly impacts the development of phonological awareness skills.

Dias et al.^(26)^ used the RGDT to assess the auditory temporal resolution of individuals with APD, the effect of maturation on this skill, and the relationship between the performance of individuals with APD on the RGDT and other AP assessment tests. The subjects were divided into two groups: APD (n = 131) and subjects with normal AP (n = 94). Approximately half of the children with APD (48%) failed the RGDT, and this percentage decreased with age. The highest percentage (86%) was found in children aged 5 to 6 years (when the system is maturing). The RGDT results correlated with those of the dichotic listening tests. According to the authors, supported by the writings of Wightman^(27)^, younger children are more likely to guess the answers, justifying the varied answers and what occurred in the present study.

Binaural interaction, assessed by MLD, is responsible for the AP of different complementary information presented simultaneously to both ears.

This study found that this ability did not change statistically between M1 and M2. This lack of difference in assessment performance before and after stimulation may be associated with the auditory pathway maturation up to the lower brainstem, present in this age group^(28)^. This is the structure assessed by MLD, responsible for detecting differences in time and intensity between the ears and helping perceive acoustic signals in noise.

Van Deun et al.^(29)^ observed that the age of 4 to 5 years can be a transition period to reach the adult level of performance in the MLD test. From 5 years old, children already present responses similar to adults in the MLD, as observed in this study.

The results discussed above reinforce the need to provide high-quality auditory stimuli to young children. Children go through periods of significant brain plasticity up to 6 years old and learn to read and write before 7 years old, making it crucial to stimulate them at earlier ages and identify children with HA-related hearing difficulties early. Stimulating preschoolers’ hearing benefits their development and reduces the negative impacts of APD on communication, learning, and social skills.

Learning to read requires phonological, orthographic, and semantic processing skills. When one reads, the central nervous system transforms graphic representations into mental sound representations and associates them with their meanings^(30,31)^. Children's transition between grades in school works as a cascade of events that gradually prepares them for this major acquisition.

Concerning the acquisition of reading skills, we also need to observe the skills acquired in the domains of both forms of communication – i.e., the predictors of reading. Phonological processing (phonological awareness, lexical access, and phonological working memory) assessment in students in the last year of preschool shows this transition of events. This justifies the zero scores throughout the evaluations (M1 and M2) in the protocol used in this study, such as in phonemic skills (which are expected for elementary school) and a constant evolution in the syllabic skills of both groups. These data were confirmed by repeated measures MANOVA, which revealed, regardless of the intervention, a significant effect between M1 and M2 performances in all pre-reading and reading skills test (p < 0.01) – except for phonemic synthesis, whose percentage of correct answers was zero in both groups in the M1 and M2 assessments, when they were still in preschool.

The stimulation type (auditory or placebo) interacted with the M1 and M2 assessments (p < 0.001***).

Univariate analysis revealed statistically significant differences between the ASG’s and PG’s performances in rhyme production, rhyme identification, word production, and working memory. These findings are in line with the study by Carroll et al.^(32)^, which states that preschool phonological awareness can be divided into an initial phase (when the child is sensitive to the implicit sound similarities of words [receptive vocabulary]) and a later phase (when they becomes aware of smaller segments, the phonemes, based on previous acquisition). In other words, the development of phonological representations follows a process of refinement, from global to segmental characteristics, which is why they are found in preschoolers and in the initial phases of learning to read and write.

In this sense, this study’s program activities, by stimulating AP, seem to have contributed positively to a better discrimination and use of the syllabic segments of words, whether in the perception of such elements in the final position (by better identifying the rhyme), or in the process of reflecting on and evoking a word with a certain signaled element (rhyme identification and word production from the given phoneme). It is worth reinforcing that the study assessed word production from a given phoneme (rather than its initial grapheme) to observe the activation of the phonological processor (through the auditory pathway) and not the orthographic one since the children were still in preschool.

### Study limitations

As previously mentioned, the study plan was to assess all students’ hearing before applying behavioral tests to assess AP. It is known that a methodology with hearing assessments ensures adequate auditory sensitivity and the integrity of the tympanic-ossicular chain, even though the tests applied do not have a diagnostic purpose.

However, data collection was permeated by the COVID-19 pandemic and, unfortunately, the ideal collection periods occurred close to the red phases and the beginning of the return to classes, when the movement of people between institutions was inadvisable. Since the evaluator already had the notebook in hands for the research, and the formal audiological assessment instruments had to be removed and returned to the university (headquartered in another municipality), it was decided to apply the same sensation level to all participants. Hence, the researchers prioritized sanitary conditions and sought to minimize the impacts of this decision on the research method (which had already been greatly interfered with by the health scenario) to keep the study ongoing.

Further research can use the initial method (with formal assessments) to verify whether it obtains similar results after applying the program.

## CONCLUSION

The HA stimulation program for schools proposed in this study consists of recreational activities that must be applied daily during 25 weeks of the school year, incorporated into the curriculum, helping children develop the skills recommended by the Ministry of Education.

After auditory stimulation, participants performed better in auditory figure-ground, temporal resolution, identification, rhyme production and word production from a given phoneme.

## References

[1] Moore JK, Guan YL (2001). Cytoarchitectural and axonal maturation in human auditory córtex. J Assoc Res Otolaryngol.

[2] Jain C, Priya MB, Joshi K (2020). Relationship between temporal processing and phonological awareness in children with speech sound disorders. Clin Linguist Phon.

[3] Vanvooren S, Poelmans H, Voz AD, Ghesquère P, Wouters J (2017). Do prereaders’ auditory processing and speech perception predict later literacy?. Res Dev Disabil.

[4] Murphy CFB, Schochat E (2009). Correlações entre leitura, consciência fonológica e processamento temporal auditivo. Pró-Fono R Atual Cient.

[5] Fostick L, Revah H (2018). Dyslexia as a multi-deficit disorder: working memory and auditory temporal processing. Acta Psychol (Amst).

[6] Terto SSM, Lemos SMA (2013). Aspectos temporais auditivos em adolescentes do 6º ano do ensino fundamental. Rev CEFAC.

[7] Musiek FE, Schochat E (1998). Auditory training and central auditory processing disorders: a case study. Semin Hear.

[8] Brasil (2017). Base Nacional Curricular Comum.

[9] Pereira LD, Schochat E (2011). Testes auditivos comportamentais para avaliação do processamento auditivo central..

[10] Auditec of Saint Louis (2003). Masking Level Difference: evaluation manual.

[11] Keith RW (2000). RGDT – Random Gap Detection Test.

[12] Capellini SA, César ABPC, Germano GD (2017). Protocolo de Identificação Precoce dos Problemas de Leitura – IPPL..

[13] Hassaan MR, Ibraheem AO (2016). Auditory training program for Arabic-speaking children with auditory figure-ground déficits. Int J Pediatr Otorhinolaryngol.

[14] Masquelier MP (2003). Management of auditory processing disorders. Acta Otorhinolaryngol Belg.

[15] Tabachnick BG, Fidell LS (2007). Using multivariate statistics..

[16] Moore DR (2002). Auditory development and the role of experience. Br Med Bull.

[17] Musiek FM, Gollegly KM, Baran JA (1984). Myelination of the corpus callosum and auditory processing problems in children: theoretical and clinical correlates. Semin Hear.

[18] Baran JA, Musiek FE, Musiek FE, Rintelmann WF (2001). Perspectivas atuais em avaliação auditiva..

[19] Musiek F, Shinn J, Hare C (2002). Plasticity, auditory training, and auditory processing disorders. Semin Hear.

[20] Sharma M, Purdy SC, Kelly AS (2012). A randomized control trial of interventions in school-aged children with auditory processing disorders. Int J Audiol.

[21] Moore DR (2018). Editorial: auditory processing disorder. Ear Hear.

[22] Filippini R, Brito NFS, Neves-Lobo IF, Schochat E (2014). Maintenance of auditory abilities after auditory training. Audiol Commun Res.

[23] Cibian AP, Pereira LD (2015). Questionnaire for use in the monitoring of auditory training results. Distúrb Comun.

[24] Chermak GD, Musiek FE (2002). Auditory training: principles and approaches for remediating and managing auditory processing disorders. Semin Hear.

[25] Murphy CB, Peres AK, Zachi EC, Ventura DF, Pagan-Neves LO, Wertzner HF (2015). Generalization of sensory auditory learning to top-down skills in a randomized controlled trial. J Am Acad Audiol.

[26] Dias AMN (2004). Desempenho de escolares para o teste de detecção de intervalo de silêncio em tom puro..

[27] Wightman F, Allen P, Dolan T, Kistler D, Jamieson D (1989). Temporal resolution in subjects. Child Dev.

[28] American Academy of Audiology (2010). Clinical practice guidelines for the diagnosis, treatment and management of children and adults with central auditory processing disorder.

[29] Van Deun L, van Wieringen A, Van den Bogaert T, Scherf F, Offeciers FE, Van de Heyning PH (2009). Sound localization, sound lateralization, and binaural masking level differences in young children with normal hearing. Ear Hear.

[30] Rakhlin NV, Mourgues C, Cardoso-Martins C, Kornev AN, Grigorenko EL (2019). Orthographic processing is a key predictor of reading fluency in good and poor readers in a transparent orthography. Contemp Educ Psychol.

[31] Viterbo G, Katzir T, Goldfarb L (2020). Accelerating reading via local priming. Acta Psychol (Amst).

[32] Carroll JM, Snowling MJ, Hulme C, Stevenson J (2003). The development of phonological awareness in preschool children. Dev Psychol.

