# Supplementary material for: Hearing abilities stimulation program for schools
Source: Codas. 2025 Mar 31;37(3):e20240065. doi: 10.1590/2317-1782/e20240065en (PMC11963881; doi:10.1590/2317-1782/e20240065en)
Supplement: Translation

# Translation: Programa de estimulação das habilidades auditivas: uma proposta escolar

**DOI:** 10.1590/2317-1782/e20240065pt

**Authors:** Ana Flávia de Oliveira Nalom Baruchi, Eliane Schochat

**Affiliations:** 1 Departamento de Fonoaudiologia, Fisioterapia e Terapia Ocupacional, Faculdade de Medicina, Universidade de São Paulo – USP - São Paulo (SP), Brasil.

**Keywords:** Audição, Percepção Auditiva, Estimulação Auditiva, Leitura, Aprendizagem

## Abstract

**Objetivo:**

Elaborar e verificar a efetividade de um programa de estimulação das HA inserido na grade curricular comum e aplicado por professores em sala de aula.

**Método:**

Um programa de estimulação das habilidades auditivas (HA) foi elaborado e aplicado no ano letivo de escolares da educação infantil. Trinta e quatro crianças foram estimuladas auditivamente (GEA) e comparadas a um grupo placebo (GP. N=31). Os alunos foram avaliados quanto às HA, habilidades pré-leitoras e de decodificação, pré e pós-intervenção. Duas reavaliações foram realizadas após a aplicação do programa, permitindo um acompanhamento do desempenho dos escolares em meio à pandemia Covid-19.

**Resultados:**

O programa proposto conta com atividades que foram aplicadas pelos professores durante 25 semanas, entre 10-15 minutos/dia, em ambiente escolar. As HA estimuladas foram: detecção, discriminação, habilidades temporais, figura-fundo, fechamento, memória e atenção. Houve diferença significativa entre o desempenho do GEA e GP após a aplicação do programa.

**Conclusão:**

O programa pôde ser incorporado na grade curricular, auxiliando no desenvolvimento das habilidades preconizados pelo Ministério da Educação. Após a estimulação auditiva, percebeu-se melhor desempenho em figura-fundo auditiva, resolução temporal, identificação e produção de rima e produção de palavra a partir de fonema dado no GEA.

## INTRODUÇÃO

Os primeiros anos de vida da criança são marcados por importantes aquisições auditivas e linguísticas. Durante este período o sistema nervoso central está se moldando e se modificando a todo instante. É o que, resumidamente, conhecemos por plasticidade neural. Nesta fase, as experiências auditivas oferecidas às crianças serão fundamentais para um desenvolvimento satisfatório, pois será o ambiente (e os estímulos contidos nele) que modulará e aumentará a atividade do nervo auditivo, permitindo, ao longo dos anos, que a criança alcance uma percepção de fala satisfatória^(1)^.

Segundo Jain e colaboradores^(2)^, as habilidades de processamento temporal são fundamentais nos sete anos iniciais da vida de uma criança e desempenham um papel importante no conhecimento dos sons da fala (discriminação de vogais semelhantes e outros sons linguísticos) e na aquisição/desenvolvimento da linguagem. Neste sentido, tais habilidades estão relacionadas à capacidade de identificar e manipular segmentos de fala, ou seja, consciência fonológica.

Vanvooren e colaboradores^(3)^, em um estudo longitudinal, acompanharam 87 crianças de cinco anos de idade, com alto e baixo risco familiar para dislexia, durante o processo de alfabetização. Os autores observaram uma relação positiva entre o desempenho das habilidades auditivas (HA) na pré-escola e o posterior desempenho em fonologia e na alfabetização. Ou seja, enquanto o processamento dos sinais temporais da fala está ligado a habilidades fonológicas, o fechamento auditivo foi um preditor da alfabetização.

Outros estudos^(4,5)^ apontam uma associação entre o processamento auditivo temporal e os transtornos de linguagem oral e/ou escrita. Segundo esses autores, tais dificuldades auditivas podem acontecer dentro do espectro da fala e interferirem na formação das representações fonológicas, o que refletiria na associação grafofonêmica. Desta forma, o funcionamento adequado do sistema auditivo é um pré-requisito para um efetivo processo de ensino-aprendizagem^(6)^.

A estimulação das HA pode promover melhora na eficiência do sistema auditivo. O objetivo das atividades de estimulação auditiva é fortalecer o sistema nervoso auditivo central e os sistemas relacionados, modificando o comportamento da criança diante dos conteúdos que chegam até ela por meio da via auditiva nas tarefas cotidianas (incluindo tarefas acadêmicas)^(7)^.

A Base Nacional Comum Curricular (BNCC) preconiza que, na Educação Infantil, os alunos sejam estimulados e se tornem capazes de identificar sons produzidos por diferentes objetos e reconheçam suas qualidades (intensidade, duração, altura e timbre), de modo a se prepararem para a etapa seguinte^(8)^.

Com base no exposto, a relevância desta pesquisa justifica-se e diferencia-se de outros estudos por incorporar atividades de natureza auditiva na grade curricular comum, de maneira a auxiliar os alunos a se apropriem do sistema de escrita alfabética de modo articulado ao desenvolvimento das HA, como preconizado pela BNCC. Tudo isso, com baixo custo às instituições de ensino, lançando-se mão de materiais disponíveis em unidades educacionais. Neste sentido, o objetivo deste estudo foi elaborar e verificar a efetividade de um programa de atividades lúdicas para a estimulação das HA, inserido na grade curricular comum de alunos do último ano do Ensino Infantil executado por professores, em sala de aula e verificar seu impacto em habilidades pré-leitoras das crianças estimuladas.

## MÉTODO

Estudo longitudinal, prospectivo, analítico e intervencional, aprovado pelo Comitê de Ética em Pesquisa da Faculdade de Medicina da Universidade de São Paulo (protocolo nº 3.469.029).

O responsável pela instituição participante assinou o Termo de Autorização da Unidade Educacional Envolvida. Os professores e os pais dos escolares participantes deste estudo assinaram Termo de Consentimento Livre e Esclarecido (TCLE). Os escolares assinaram um Termo de Assentimento.

Os dados deste estudo foram coletados em uma escola pública municipal de ensino regular de uma cidade do interior de São Paulo (Brasil) e foram armazenados no Laboratório de Investigação Fonoaudiológica em Neuroaudiologia do Curso de Fonoaudiologia da Faculdade de Medicina da Universidade de São Paulo.

A sala escolhida para as avaliações apresentava o menor nível de pressão sonora da escola, com valor próximo ao permitido para o ruído ambiental das instituições segundo a Associação Brasileira de Normas Técnicas (ABNT). Para a medição do nível médio de ruído ambiental, utilizou-se o medidor de nível de pressão sonora da marca *Instrutherm* – modelo DEC-460, acatando suas instruções de uso. O ruído médio observado foi de 65 dB. Além disso, considerou-se a demanda pedagógica e a ausência de fluxo de pessoas nestes espaços.

Os critérios de inclusão desse estudo foram: estar cursando, regularmente, o último ano do Ensino Infantil (EI) da escola selecionada; ausência de indicadores de alterações de audição (histórico de otites de repetição e/ou perda auditiva familiar) e/ou visão, ausência de histórico familiar ou sinais e sintomas de alterações neurológicas, comportamentais e/ou cognitivas (triados por meio dos questionários).

Participaram deste estudo 65 crianças matriculadas no último ano do EI da referida escola, as quais obtiveram 75% ou mais de frequência nas aulas no ano letivo de aplicação das atividades, divididas em dois grupos:

- **Grupo placebo (GP):** 31 alunos os quais receberam estimulação placebo das habilidades visuais e psicomotoras durante 10-15 minutos diários, por 25 semanas do ano letivo de 2019;
- **Grupo estimulação auditiva (GEA):** 34 alunos os quais receberam estimulação auditiva durante 10-15 minutos diários, por 25 semanas do ano letivo de 2019.

A saber, as crianças pertenciam a quatro turmas da mesma escola. Foram sorteadas, randomicamente, duas turmas para comporem o GP e duas turmas para comporem o GEA.

Três etapas foram programadas para a conclusão do estudo: avaliação inicial; elaboração e aplicação do programa de estimulação das HA; e reavaliação.

### Avaliações inicial e final

Realizou-se duas avaliações: M1 (avaliação inicial); M2 (reavaliação após aplicação do programa). Aplicou-se provas para avaliação das HA e habilidades pré-leitoras e de leitura, conforme descritas pelos manuais dos testes.

Inicialmente, previu-se a avaliação auditiva de todos os escolares (maiores informações no tópico Limitações do Estudo). No entanto, por intercorrências/imprevistos durante o período da pesquisa, a qual foi transpassada pela pandemia Covid-19, impossibilitando a logística de constantes transportes dos equipamentos entre a cidade do referido laboratório e a cidade da escola parceira, não foi possível realizá-la. Dessa forma, considerando-se que avaliação das habilidades auditivas não apresentavam fins diagnósticos, mas auxiliariam na observação do desempenho das crianças pré e pós estimulação das HA, aplicou-se os testes comportamentais mantendo o nível de apresentação dos estímulos igual para todos os sujeitos (descritos adiante). A comparação dos resultados foi realizada intergrupos e inter sujeitos (de forma que as normalidades dos testes, utilizadas para avaliação formal, em cabina acústica, após identificação dos limiares auditivos individuais, não foram utilizadas. Esta foi uma opção dos autores, considerando-se que a faixa etária avaliada foi inferior à normalidade dos testes, não sendo possível comparar valores esperados à idades diferentes).

Neste processo, uma criança apresentou dificuldades em executar os testes no nível estabelecido, sendo encaminhada para avaliação auditiva formal, a qual identificou perda auditiva neurossensorial de grau leve em OD e perda auditiva neurossensorial de grau moderado em OE. Esta criança pôde participar do programa com os demais alunos, mas não foi inclusa nas avaliações do grupo GEA.

Realizou-se inspeção do meato auditivo externo de todas as crianças previamente a todas as aplicações da triagem do PA. A triagem do PA avaliou as respostas comportamentais frente aos testes:

1. Dicótico de Dígitos^(9)^ (TDD) para habilidade de integração binaural, o qual envolveu a identificação de quatro números diferentes apresentados simultaneamente nas duas orelhas, solicitou-se a repetição dos quatro números ouvidos.
2. *Masking Level Difference*^(10)^ (MLD) o qual avaliou a interação binaural, em condição binaural, a partir da apresentação de 33 segmentos de ruído de banda estreita em uma orelha, por pelo menos três segundos, na presença ou não de tom puro de 500 Hz. Três condições foram consideradas: tom puro e ruído de banda estreita em fase nas duas orelhas (condição sinal/ruído homofásica - SoNo); tom puro em fase invertida em uma das orelhas e ruído em fase nas duas orelhas (condição sinal/ruído SπNo); ruído sem a presença de tom puro (*no tone* – NT). As crianças foram orientadas a levantarem a mão toda vez que ouvissem o apito (tom puro). Para análise, foram somadas as vezes em que os participantes sinalizaram ouvir um som. Em seguida, este número foi convertido em dB, de acordo com o quadro disponível no teste.
3. *Random Gap Detection Test*^(11)^ (RGDT – versão padrão) para habilidade de resolução temporal, no qual as crianças foram submetidas a apresentação de tom puro, nas frequências de 500, 1000, 2000 e 4000 Hz, na condição binaural. Os tons puros pareados apresentaram tempo de intervalo e com variação de zero a 40 ms em ordem aleatória, com incrementos que variam de dois a 10 ms. As crianças foram orientadas, a cada apresentação, a apontar (em uma imagem onde continham um ou dois quadrados [■ ou ■■]) ou dizer se estavam ouvindo um ou dois sons.

Para todos os testes, aplicados com auxílio de *notebook* e fone (Sony MDR-7506 com fio) inspecionado, foram realizadas etapas treino. Para avaliar o nível de apresentação dos estímulos foi utilizado o equipamento audiodosímetro SV-102®, da marca Svantek®, no qual foi plugado um microfone nas saídas dos fones utilizados, com o computador em seu volume médio. Os valores médios do nível de pressão sonora (NPS) capturados pelo instrumento foram: TDD – 58dB; MLD – 58,1 dB; RGDT – 500Hz: 54,1 dB | 1000Hz: 54,1dB | 2000 Hz: 57,7dB | 4000Hz: 51,4dB.

Com o objetivo de eliminar o viés “ordem de aplicação” nos resultados e na interpretação dos testes da triagem do PA, foram utilizadas as duas sequências de aplicação dos testes (TDD + RGDT + MLD ou MLD + RGDT + TDD). 50% dos sujeitos de cada grupo foram inclusos em cada sequência.

O tempo médio para aplicação dos testes auditivos foi de 40 minutos, considerando-se a necessidade de explicações adequadas para a idade e nível de compreensão verbal dos alunos.

O Protocolo de Identificação Precoce dos Problemas de Leitura, proposto por Capellini e colaboradores^(12)^ foi escolhido para a avaliação de habilidades pré-leitoras e de leitura. Não foram aplicados todos os itens do protocolo, uma vez que as crianças do EI ainda não haviam adquirido todas as habilidades a serem avaliadas pelo instrumento. No entanto, buscou-se aplicar o exemplo de todas as habilidades com o objetivo de identificar em qual das avaliações uma nova habilidade pôde ser observada. O tempo médio de aplicação deste protocolo foi de 25 minutos.

As habilidades avaliadas nesta pesquisa foram:

- Identificação de letras a partir da apresentação das mesmas ao aluno para que este identificasse o nome da letra e o valor sonoro;
- Produção de rima a partir da apresentação auditiva de 20 palavras para que o mesmo dissesse uma palavra que terminasse com o mesmo som;
- Identificação de rima a partir da apresentação auditiva de 20 trios de palavras para identificação das palavras terminadas com o mesmo som.
- Segmentação silábica a partir da apresentação auditiva de 21 palavras a serem separadas por sílabas.
- Produção de palavras a partir dos fonemas do alfabeto;
- Memória operacional fonológica a partir da repetição de 24 não palavras;
- Nomeação automática rápida de sete sequências intercaladas de desenhos coloridos (carro, bola, pato, casa e chave);
- Atenção visual a partir da leitura silenciosa duplas de palavras para associação com uma figura-alvo;
- Leitura em voz alta de 20 palavras e 20 não palavras;
- Compreensão de 20 frases incompletas com figuras ilustrativas associativas apresentadas para que o aluno pudesse completar oralmente as frases.

A avaliação das habilidades leitoras foi realizada em um dia diferente ao das HA, no mesmo ambiente de aplicação dos testes que avaliaram o PA.

### Elaboração do programa de estimulação das habilidades auditivas

Para a elaboração do conjunto de atividades que compõem o programa, considerou-se as HA de detecção, discriminação, figura-fundo, fechamento, habilidades temporais (discriminação dos padrões de frequência, duração e intensidade), memória e atenção. Indiretamente, ao longo do ano as tarefas estimularam a localização sonora.

Buscou-se respeitar a ordem natural de aquisição e desenvolvimento das HA, o grau de compreensão esperado para a faixa etária estudada, materiais de fácil acesso para a escola pública participante (previamente consultados pela pesquisadora na escola participante), compreensão dos professores acerca das atividades a serem aplicadas, bem como sobre a importância dos objetivos delas. Além disso, considerou-se que organização e configuração das salas de aulas eram diferentes. A fim de minimizar este impacto e permitir que os escolares recebessem os estímulos-alvo vindos de diferentes localizações, orientou-se aos professores que janelas e portas da sala deveriam permanecer fechadas durante a aplicação do programa e que os alunos deveriam se sentar ou o professor se posicionar em diferentes localizações.

Quando os professores identificavam que as crianças não compreendiam a tarefa, imediatamente a pesquisadora era acionada por meio da vice-diretora escolar. As instruções eram modificadas ou uma adaptação para a atividade era oferecida. Considerou-se atividade adequada quando os dois professores aplicadores indicavam pontuação geral entre 8 e 10 (na avaliação presente no verso da explicação semanal entregue aos mesmos).

As atividades do Programa basearam-se na adaptação de estratégias utilizadas no treinamento auditivo realizado na prática clínica fonoaudiológica, bem como em estratégias utilizadas em pesquisas de treinamento auditivo realizadas em contexto escolar^(13,14)^.

A adaptação sugerida para este Programa visou: a participação em grupo nas atividades; o fato de que algumas crianças poderiam utilizar outras vias sensoriais de apoios (como a via visual); o benefício de, mesmo utilizando a via visual, aprender com o estímulo auditivo e a imitação dos pares; as diferenças presentes no ambiente físico de cada sala de aula; a precocidade em que os estímulos estariam sendo apresentados no contexto escolar; os benefícios dos professores poderem conhecer, semana a semana, uma nova habilidade auditiva e observá-la na prática; a compreensão de que os materiais podem variar, no entanto as estratégias devem influenciar o funcionamento do SNAC.

O tempo de aplicação das atividades auditivas diárias foi de 10 a 15 minutos. Estipulou-se que as crianças do grupo placebo executariam as atividades visuais ou psicomotoras diariamente considerando-se o mesmo tempo (10-15 minutos).

Buscando realizar um estudo duplo-cego, quinzenalmente a pesquisadora orientava verbalmente e treinava um grupo de quatro professoras (duas aplicadoras das atividades auditivas e duas aplicadoras das atividades placebo) ao mesmo tempo, sobre ambas as estimulações. Caso tivessem dúvidas, deveriam solicitar que a vice-diretora entrasse em contato com a pesquisadora (via telefone). Esta atuou como intermediária entre professoras e pesquisadora.

Embora não fosse objetivo deste estudo avaliar a evolução de cada sujeito diariamente, a cada semana os professores eram convidados a avaliar a atividade proposta a nível de sala de aula. Considerando-se o diálogo semanal com a equipe escolar, embora houvesse um cronograma de habilidades a serem estimuladas, as atividades foram sendo pensadas conforme os desafios encontrados.

Análises descritivas dos resultados dos testes comportamentais por meio da construção de tabelas com valores observados das estatísticas descritivas (média, desvio padrão, por grupo e por orelha) foram realizadas por um profissional estatístico.

Para comparar as médias das variáveis estudadas nos dois grupos (GEA e GP), nos períodos estudados, foram aplicadas as técnicas de análise multivariada de variância (MANOVA) e análise multivariada de variância com medidas repetitivas (*repeated measures* MANOVA), respectivamente. Na MANOVA, o p-valor e a razão F (que é utilizada para testar a diferença global entre grupos) foram analisados por meio do teste Lambda de Wilks (λ de Wilks)^(15)^.

Para complementar a análise descritiva, utilizamos o Intervalo de confiança de 95% e um nível de significância (p) de 0,05 (5%).

## RESULTADOS

### Programa de estimulação das habilidades auditivas

A versão final do Programa contém atividades que contemplam 25 semanas do ano letivo e estimulam as HA de detecção, discriminação, figura-fundo auditiva, fechamento, habilidades temporais (discriminação dos padrões de frequência, duração e intensidade), memória e atenção.

A média de tempo de aplicação das atividades variou entre dez e 15 minutos/dia. Todas as atividades foram aplicadas entre 80 e 100% dos dias sugeridos.

No Quadro 1, é possível observar uma lista final contendo todos os recursos utilizados, bem como um resumo das atividades propostas pelo Programa, de acordo com o objetivo ou habilidade alvo estipulada para cada semana.

**Quadro 1:** Lista de recursos utilizados e resumo das atividades propostas de acordo com os objetivos finais

Semana	Habilidade alvo	Recursos utilizados	Resumo da atividade
1	Detecção auditiva	- Sino, reco-reco, chocalho, tambor, triângulo, palmas, voz do professor	- Apresentação do instrumento;
			- Identificar e levantar a mão ao ouvir um som no silêncio;
2			Apresentação de 20 estímulos.
3	Memória auditiva	- Tambor, chocalho, reco-reco, sino e coco	- Identificação dos instrumentos isolados;
			- Memorização e identificação de 14 sequências sonoras instrumentais com um, dois, três ou quatro estímulos.
4	Atenção	-Aparelho reprodutor sonoro;	- Bater palma ao ouvirem a palavra-alvo de cada música tocada;
		-Canal do YouTube “Galinha Pintadinha”;	- Duas músicas diárias.
		-Músicas selecionadas.	
5	Discriminação	- Cartaz com figuras iguais e diferentes;	- Conceito de igual e diferente;
		- Guizo, coco, chocalho e triângulo;	- Identificar se os sons tocados eram iguais ou diferentes, utilizando recurso visual de apoio;
6		- Voz do professor e lista de palavras.	-Apresentação de 10 a 15 sequências.
7	Memória	- Figuras dos campos semânticos escolhidos pelo professor (se necessário).	- Brincadeira “fui à feira...”;
			- 10 minutos.
8	Atenção	- Coleção Estrelinha I (Sônia Junqueira, 2019).	- Explicação da atividade;
			- Levantar a mão toda vez que a palavra-alvo de uma história da Coleção Estrelinha I fosse lida;
			- Roda de conversa e compreensão da história para completar 15 minutos.
9	Percepção do padrão de duração sonora	- Instrumentos musicais selecionados pelo professor;	- Apresentação do instrumento do dia;
		- Cartaz com recurso visual para conceito de curto e longo;	- Identificação da diferença entre som curto e som longo;
		- Lista com a sequência de estímulos.	- Nomear as 10 sequências apresentadas;
10	Percepção do padrão de frequência sonora	- Tambor, triângulo, sino e coco.	- Apresentação dos instrumentos do dia;
		- Lista com a sequência de estímulos.	- Desenvolver o conceito de som fino (agudo) e grosso (grave) e associar aos instrumentos do dia;
			- Nomeação das 10 sequências apresentadas;
11	Memória	- Lista de palavra-alvo;	- Memorizar sequência de palavras e evocá-las, de tempo e tempo, durante a aula;
		- Relógio.	-Intervalo entre as evocações entre 40 e 60 minutos.
12	Atenção	-Aparelho reprodutor sonoro;	- Explicação da atividade;
		-Canal do YouTube “Palavra Cantada”;	- Bater palma sempre que ouvir a palavra-alvo;
		-Músicas selecionadas.	-Duas músicas por dia.
13	Figura-fundo	-Aparelho reprodutor sonoro;	- Ouvir a história contada pelo professor (entre 110 e 140 palavras) enquanto um ruído de fundo (musical) era apresentado simultaneamente;
		-Canal do YouTube “Palavra Cantada”;	- Roda de conversa e compreensão leitora até completar 15 minutos.
		-Músicas selecionadas;	
14		- História selecionada pelo professor.	
15	Memória	- Lista de palavras-alvo;	- Memorizar sequência de palavras e evocá-las, de tempo e tempo, durante a aula;
		- Relógio.	-Intervalo entre as evocações entre 40 e 60 minutos.
16	Atenção	- Textos selecionados pelo professor.	- Ouvir a história contada pelo professor (entre 110 e 140 palavras) e levantar a mão ao ouvir a palavra-alvo;
			- Roda de conversa e compreensão leitora até completar 15 minutos.
17	Fechamento	- Músicas selecionadas pelo professor;	- Ignorar a presença das músicas sendo tocadas durante 15 minutos das atividades da rotina escolar.
18		- Aparelho reprodutor sonoro.	
19	Memória	- Figuras dos campos semânticos escolhidos pelo professor (se necessário).	- Brincadeira “fui à feira...” por 15 minutos.
20	Atenção	- Utensílios selecionados pelo professor;	- Apresentação dos quatro objetos do dia aos alunos;
			- Identificação dos sons dos objetos.
			- Nomeação das 15 sequências com três sons cada tocadas pelo professor;
			- Identificação do som ausente na sequência tocada.
21	Detecção	- Lista de estímulos linguísticos;	- Levantar a mão e permanecer com ela levantada enquanto ouvirem 20 sequências com estímulos silábicos ou fonêmicos alvo.
		- Voz do professor.	
22	Discriminação	- Figuras selecionadas pelo professor para reforçar o conceito de igual e diferente;	- Discriminação de palavras ou pares mínimos;
		- Lista de palavras;	- 10 sequências diárias.
		-Voz do professor.	
23	Percepção do padrão de intensidade sonora	- Chocalho, sino, guizo, coco e tambor;	- Apresentação dos instrumentos do dia;
		- Lista de apresentação dos estímulos.	- Identificar a diferença entre som alto e som baixo (de acordo com a intensidade);
			- Nomear as 10 sequências apresentadas;
24	Figura-fundo	- Som competitivo selecionado pelo professor (música desconhecida, instrumental, rádio mal sintonizado).	- Brincadeira “Fui à feira...” ao mesmo tempo em que um ruído era apresentado.
			- 10 minutos de atividade.
25	Fechamento	- Lista de grafemas ou figuras elaborada pelo professor;	- Ditado de 15-20 grafemas/dia simultâneo à apresentação de som competitivo.
		- Orientação de adaptação da atividade;	
		- Músicas selecionadas pelo professor.	

### Efetividade da aplicação do Programa de estimulação das habilidades auditivas no Ensino Infantil

Com relação à idade dos indivíduos, em M1 a faixa etária média foi de 60 meses. Em M2, essa média avançou para 68 meses. Pela ANOVA, não foi detectada diferença estatisticamente significante entre as médias das idades em nenhum dos momentos avaliados e nem diferença entre os grupos: M1 [F(1,63)=0,24, p=0,63] e M2 [F(1,63)=0,24, p=0,63].

Na comparação quanto ao sexo dos participantes do estudo, o teste qui-quadrado não mostrou associação entre grupo e gênero *X* ^2^(1) = 0,42, (p=0,52).

A Tabela 1 apresenta os valores médios e desvios-padrão do desempenho de GEA e GP nos testes de processamento auditivo (TDD, MLD e RGDT) na avaliação inicial (M1) e após a intervenção (M2).

**Tabela 1:** Performance dos grupos nos testes de processamento auditivo, nos períodos pré e pós-intervenção (M1 e M2)

			**GEA**				GP			
			M1		M2		M1		M2	
			**Média**	**Desvio padrão**	**Média**	**Desvio padrão**	**Média**	**Desvio padrão**	**Média**	**Desvio padrão**
TDD	OD		53,53	13,07	72,79	15,53	53,55	13,05	60,48	12,67
	OE		52,13	16,82	72,06	16,77	50,76	13,66	59,71	14,86
MLD			7,88	3,75	6,53	4,22	9,19	5,63	6,90	4,06
RGDT			11,14	4,40	9,74	3,61	14,65	6,90	13,95	4,94

**Legenda:** GEA – Grupo Estimulação Auditiva; GP - Grupo Placebo; OD – orelha direita, OE – orelha esquerda; M1 – Momento um; M2 – Momento dois; TDD – Teste Dicótico de Dígitos; MLD – *Masking Level Difference*, RGDT - *Random Gap Detection Test*

A MANOVA de medidas repetidas revelou que houve um efeito estatisticamente significante entre a performance observada nos períodos M1 e M2 [F (4,60) = 22,12, p<0,001***, η^2^ parcial=0,60, λ de Wilks=0,40]. A análise univariada revelou que houve diferença estatisticamente significante entre o M1 e M2 em todos os testes do PAC (p<0,05).

Além disso, a análise revelou uma interação significativa entre os períodos (M1 e M2) e o tipo de intervenção realizada (GEA e GP): [F (4,60) = 4,10, p=0,005**, η^2^ parcial=0,22, λ de Wilks=0,78].

As diferenças estatisticamente significantes entre as performances de GEA e GP ocorreram nas variáveis TDD OD (p=0,002**) e TDD OE (p=0,008**).

A interação entre período e grupo foi adicionalmente investigada com o uso do teste t. Dado que existem dois testes de efeito simples, o critério de significância foi ajustado para 0,025.

Para o GEA, as análises mostraram que houve diferença estatisticamente significante no desempenho entre os momentos pré e pós-intervenção para o TDD – OD [t(33)=-6,76, p<0,001***], TDD – OE [t(33)=-7,31 p< 0,001***] e RGDT [t(33)=-6,76, p<0,001***]. Entretanto, não foi observada diferença estatisticamente significante no MLD [t(33)=1,54, p= 0,132].

Para o GP, houve diferença estatisticamente significante no desempenho entre os momentos pré e pós-intervenção para o TDD – OD [t(30)=-2,80, p=0,009**] e TDD – OE t(30)=-2,99 p< 0,005**]. No GP, não houve diferença estatisticamente significante nos testes MLD [t(30)=2,06, p=0,04] e RGDT [t(30)=0,57, p= 0,567].

A Tabela 2 apresenta os valores médios e desvios-padrão do desempenho de GEA e GP nos testes das habilidades pré-leitoras e de leitura na avaliação inicial (M1) e após a intervenção (M2). É importante notar que, para essa análise, houve a redução do número de indivíduos no GP, uma vez que duas crianças não concluíram a reavaliação.

**Tabela 2:** Performance dos grupos nos testes de habilidades pré-leitoras e decodificação, nos períodos pré e pós-intervenção (M1 e M2)

	GEA (n=34)					GP (n=29)					
	M1		M2			M1			M2		
	Média	DP		Média	DP		Média	DP		Média	DP
Número de acertos no teste de identificação de letras	78,40	21,37		97,06	5,32		65,37	29,30		88,01	24,31
Número de acertos no teste de produção de rima	0,00	0,00		11,32	17,94		0,00	0,00		3,62	7,43
Número de acertos no teste de identificação de rima	0,00	0,00		50,74	31,00		0,00	0,00		20,86	23,98
Número de acertos no teste de segmentação silábica	18,05	35,04		88,00	13,59		0,00	0,00		62,29	28,25
Número de acertos no teste de produção de palavras a partir de fonema dado	31,10	27,11		69,74	23,13		42,60	30,36		57,60	31,40
Número de acertos no teste de memória operacional fonológica	75,97	13,30		92,46	9,41		73,95	25,25		83,14	18,38
Tempo, em segundos, no teste de nomeação automática rápida	57,03	11,09		46,12	10,90		49,76	16,45		44,48	9,67
Número de acertos no teste de leitura silenciosa	55,29	19,73		76,18	17,76		56,21	24,56		64,48	21,97
Número de acertos no teste de leitura de palavras e pseudopalavras	0,29	1,72		5,59	16,04		0,00	0,00		4,74	13,03
Número de acertos no teste de compreensão auditiva de sentenças	68,53	10,48		83,38	17,00		68,62	23,68		79,48	15,77
Número de acertos no teste de síntese fonêmica	0,00	0,00		0,00	0,00		0,00	0,00		0,00	0,00

**Legenda:** GEA – Grupo Estimulação Auditiva; GP - Grupo Placebo, n – número; M1 – Momento 1; M2 – Momento dois; DP - desvio padrão

A MANOVA de medidas repetidas revelou que houve um efeito significativo entre a performance observada nos períodos M1 e M2 [F (10,52) = 87,79, p<0,001***, η^2^ parcial=0,94, λ de Wilks=0,05]. A análise univariada revelou que houve diferença estatisticamente significante entre o M1 e M2 em todos os testes das habilidades pré leitoras e de leitura (p<0,01), exceto para a prova de síntese fonêmica, no qual a porcentagem de acertos foi ‘zero, em ambos os grupos, tanto para a avaliação M1 quanto para M2.

As diferenças estatisticamente significantes entre as performances de GEA e GP ocorreram em quatro variáveis, a saber: Produção de Rima (p=0,03*), Identificação de rima (p<0,001***), Produção de Palavra (p<0,001***) e Memória Operacional (p=0,04*).

A interação entre período e grupo foi adicionalmente investigada com o uso do teste t, com o ajuste no critério de significância para 0,025.

Para o GEA, as análises mostraram que houve diferença estatisticamente significante no desempenho entre os momentos pré e pós-intervenção para as prova de: ‘Compreensão Alfabética’ [t(33)=-5,77, p<0,001***], Produção de Rima [t(33)=-3,68, p=0,001***], Identificação de rima [t(33)=-9,55 p<0,001***], ‘Segmentação Silábica’ [t(33)=-12,15, p<0,001***], Produção de Palavra [t(33)=-8,71, p<0,001***], Memória Operacional [t(33)=-10,78, p<0,001***], ‘Nomear Estímulos’ [t(33)=7,38, p<0,001***], ‘Leitura Silenciosa’ [t(33)=-5,55, p<0,001***], e ‘Compreensão Auditiva’ [t(33)=-4,83, p<0,001***]. Não foi observada diferença estatisticamente significante na prova de ‘Leitura Oral’ [t(33)=-1,94, p=0,06].

Para o GP, houve diferença estatisticamente significante no desempenho entre os momentos pré e pós-intervenção para as prova de: ‘Compreensão Alfabética’ [t(28)=-4,63, p<0,001***], ‘Produção de Rima’ [t(28)=-2,62, p=0,01**], Identificação de rima [t(28)=-4,68 p< 0,001***], ‘Segmentação Silábica’ [t(28)=-11,87, p<0,001***], Produção de Palavra [t(28)=-3,23, p=0,003**], Memória Operacional [t(28)=-2,79, p=0,009**] e ‘Compreensão Auditiva’ [t(28)=-3,22, p=0,003**]. No GP, não foi observada diferença estatisticamente significante nas provas de ‘Nomear Estímulos’ [t(28)=1,54, p=0,13], ‘Leitura Silenciosa’ [t(28)= -1,47, p=0,15] e ‘Leitura Oral’ [t(28)=-1,95, p=0,06].

## DISCUSSÃO

### Programa de estimulação das habilidades auditivas

O principal objetivo do presente estudo foi elaborar e, em seguida, verificar a efetividade de um Programa de estimulação HA, que pudesse ser aplicado por professores em ambiente escolar e incorporado à grade curricular comum, haja vista a importância da estimulação precoce das HA (fortemente associadas a posterior alfabetização).

No que se refere ao processo de criação do Programa proposto neste estudo, buscou-se elaborar atividades que pudessem ser aplicadas de maneira informal, em ambiente escolar, com baixo custo para as instituições que viessem aproveitar este material futuramente.

O programa pode ser considerado informal, pois utiliza estímulos acusticamente não controlados, materiais simples apresentados ao vivo por professores orientados a partir de um manual.

Embora o objetivo do Programa proposto não fosse treinar sujeitos com TPA, as orientações pertinentes às diferentes abordagens frente ao TPA descritas pela ASHA foram consideradas. Por este motivo, escolheu-se trabalhar com todas as HA ao longo do ano (remediação direta de habilidades); estratégias compensatórias foram consideradas no processo de aquisição/desenvolvimento das habilidades estimuladas em cada atividade; e, por fim, a gama de modificações ambientais possíveis dentro da sala de aula (onde há uma dinâmica circulação dos alunos, professores e objetos presentes no espaço) foram utilizadas.

Segundo Masquelier^(14)^, esses três métodos se complementam para fornecer treinamento auditivo *bottom-up,* juntamente com o recrutamento de funções cerebrais de ordem superior, ou seja, a abordagem *top-*down. Mudar o ambiente de apresentação dos estímulos pode maximizar as oportunidades de processar estímulos auditivos de forma eficaz, uma vez que se aproximam das situações comuns, onde as habilidades são recrutadas a todo momento.

O modelo final do Programa foi estruturado para ocorrer durante 25 semanas do ano letivo, cinco vezes por semana, numa média de tempo de 10-15 minutos de atividades diárias, ou seja, um período de tempo curto, para não prejudicar os conteúdos preconizados pelo MEC e programados pelos professores, porém suficiente para impactar positivamente no desenvolvimento dos escolares. As atividades realizadas englobaram as HA de detecção, discriminação, percepção dos padrões de duração, frequência e intensidade, figura-fundo, fechamento, atenção e memória.

Considerando-se que as atividades propostas foram aplicáveis aos escolares do último ano do EI, sugere-se que sejam testadas e, se necessário, adaptadas para os alunos do 1º ano do EF.

### Efetividade da aplicação do Programa de estimulação das habilidades auditivas no Ensino Infantil

De maneira geral, os resultados indicaram que houve um melhor desempenho de ambos os grupos com o avanço das avaliações, como esperado para o desenvolvimento infantil, e que o GEA apresentou melhores desempenhos se comparado ao GP após a estimulação auditiva.

A análise de variância não detectou diferença estatisticamente significante entre as médias das idades em nenhum dos momentos avaliados. Todas as crianças da pesquisa encontraram-se dentro da idade ideal (segundo o Ministério da Educação – MEC) para cursarem o último ano da Educação Infantil.

Não foram observadas associações entre os grupos, gênero e idade (p=0,52), demonstrando que há um equilíbrio entre meninos e meninas em ambos os grupos.

Ambos os grupos obtiveram melhores resultados em todos os testes aplicados em M2, se comparados os resultados no M1. Esse fato pode ser justificado pelo período do desenvolvimento em que as crianças do estudo se encontravam. Os primeiros anos de vida de uma criança são fundamentais para que o SNAC continue o desenvolvimento iniciado ainda em vida intrauterina e período neonatal^(16)^. Durante os primeiros anos da infância observa-se maior plasticidade do SNA e a maturação deste sistema, permitindo o estabelecimento de novas conexões neurais^(17,18,19)^.

Houve, segundo a MANOVA, uma interação significativa entre os períodos M1 e M2 e o tipo de intervenção realizada (GEA apresentou resultados superiores em M2, se comparados ao GP). Neste sentido, podemos afirmar que a apresentação de estratégias de estimulação das HA, em sala de aula, de maneira lúdica e contínua (por um período curto de tempo em cada dia, sem que os professores necessitassem retirar nenhuma atividade de seu planejamento anual), favorece de maneira positiva as habilidades trabalhadas, reforçando o fato das experiências auditivas positivas oferecidas à criança serem ferramentas fundamentais para o bom desenvolvimento e aprimoramento da eficiência do sistema perceptual auditivo^(1,16,20,21)^, o qual impacta diretamente no desenvolvimento da consciência fonológica.

Para o GEA, as análises mostraram que houve diferenças estatisticamente significante no desempenho entre M1 e M2 para a habilidade de figura-fundo auditiva e resolução temporal. A habilidade auditiva de figura-fundo para sons linguísticos é fundamental em ambientes comunicativos que envolvem a tarefa de direcionar a atenção ao estímulo alvo em competição com outros estímulos de menor relevância. Em sala de aula, por exemplo, o aluno necessita direcionar a atenção para a explicação do professor e compreender uma mensagem dita, embora muitas vezes o ambiente esteja ruidoso.

Semelhante aos resultados da presente pesquisa, outros estudos analisaram o desempenho de alunos quanto a habilidade de figura-fundo após treinamento auditivo e mostraram uma evolução desta habilidade concomitantemente a outras HA (alteradas ou não)^(22,23)^.

Estudos que adotaram o treinamento de dessensibilização ao ruído para aprimorarem a percepção de fala revelaram que esta tarefa influencia positivamente a habilidade de figura-fundo simulando demandas da vida real^(3,21)^.

Semelhante à atual pesquisa, Hassaan e Ibraheem^(13)^ sugeriram um programa de treinamento auditivo da habilidade de figura fundo cujo material (em língua árabe) objetivou a dessensibilização com o uso de um ruído apresentado simultaneamente à uma história (lida pelo professor), considerando os desafios dentro das salas de aula.

Após ouvirem as histórias, os sujeitos eram convidados a responder perguntas, demonstrando o quanto eram capazes de compreender e memorizar durante a tarefa, expandindo os períodos de atenção auditiva e a memória, que são críticas para o aprendizado (na atual pesquisa, devido à idade das crianças, a roda de conversa sobre o texto, com auxílio do professor no processo de reconstrução da história, substituiu questionários formais). Segundo os autores, esta forma de apresentação dos estímulos combinou a flexibilidade dos programas informais e a capacidade de modificação do funcionamento cortical dos programas formais (ainda que de maneira menos intensa), uma vez que a habilidade-alvo das crianças foi aprimorada.

Chermak e Musiek^(24)^ relataram que tarefas auditivas complexas requerem discriminação de eventos acústicos, além de processamento temporal auditivo e ações cognitivas como atenção e memória. Neste sentido, a estimulação auditiva objetivando a compreensão de fala diante de situações ruidosas/desfavoráveis pode ser considerada uma tarefa auditiva complexa o suficiente para fortalecer globalmente o PA, explicando o fato de programas que objetivam estimular uma habilidade específica auxiliarem no desenvolvimento ou aprimoramento de outras HA. De acordo com Murphy e colaboradores^(25)^, essa melhora não se limita às HA, mas também se estende às habilidades de memória, atenção e linguagem (justificando os resultados dos testes de leitura que serão descritos posteriormente).

Em todos os estudos citados acima, a população estudada apresentava idade superior a idade dos sujeitos da atual pesquisa. Acerca dessa lógica, podemos inferir que também a população pré-escolar tem as HA beneficiadas e, consequentemente, habilidades de memória, atenção e linguagem, diante da aplicação de tarefas que envolvam seleção de um estímulo alvo em competição a outros.

Como mencionado anteriormente, a resolução temporal das crianças estimuladas auditivamente parece ter sido beneficiada com as atividades propostas no Programa desenvolvido (ou, uma somatória da influência do processo do desenvolvimento e da plasticidade relacionada à aprendizagem diante das tarefas apresentadas). Embora o controle dos estímulos apresentados não seja extremamente preciso como em programas formais (que lançam mão de técnicas informatizadas), as atividades auxiliaram na percepção das variações acústicas dentro de um espaço de tempo (maior ou menor). Segundo Musiek et al.^(19)^, a resolução temporal relaciona-se à capacidade de perceber pequenos intervalos de silêncio dentro ou entre os segmentos da fala. Portanto, o processamento da fala depende, em parte, do processamento temporal, o qual irá impactar diretamente no desenvolvimento de habilidades de consciência fonológica.

Dias et al.^(26)^ utilizaram o RGDT para verificar a habilidade de resolução temporal auditiva em indivíduos com TPA, o efeito da maturação nesta habilidade e a relação do desempenho de indivíduos com TPA no RGDT e em outros testes que avaliam o PA. Os sujeitos foram divididos em dois grupos: TPA (n=131) e sujeitos com PA normal (n=94). Aproximadamente metade das crianças com TPA (48%) falharam no RGDT e este percentual diminuiu em função da idade. O maior percentual (86%) foi encontrado nas crianças de 5 a 6 anos (fase em que o sistema se encontra em processos de maturação). Houve correlação entre os resultados do RGDT e os testes de escuta dicótica. Segundo os autores, apoiados nos escritos de Wightman et al.^(27)^, as crianças mais novas estão mais propensas a tentar adivinhar as respostas, justificando a variabilidade das respostas assim como o ocorrido no presente estudo.

A interação binaural, avaliada pelo MLD, é responsável pelo processamento auditivo de informações diferentes que se complementam, apresentadas nas duas orelhas simultaneamente.

Observou-se, neste estudo, que tal habilidade não mudou, estatisticamente, entre M1 e M2. Essa ausência de diferença no desempenho entre as avaliações pré e pós estimulação pode estar associada à maturação da via auditiva até tronco encefálico baixo, presente nessa faixa etária^(28)^. Esta é a estrutura avaliada pelo MLD, responsável por detectar as diferenças de tempo e intensidade entre as orelhas e auxiliar na percepção do sinal acústico na presença de ruído.

Van Deun et al.^(29)^-observaram que a idade de quatro a cinco anos pode ser considerada um período de transição para atingir o nível de desempenho adulto no teste MLD. A partir dos cinco anos, as crianças já apresentam respostas semelhantes aos adultos no MLD, como observadas neste estudo.

Os resultados acima discutidos reforçam a necessidade da apresentação de estímulos auditivos de qualidade às crianças pequenas. O fato destas passarem por períodos de importante plasticidade cerebral até os 6 anos de idade e desenvolverem a alfabetização antes dos 7 anos, torna crucial a estimulação em idades anteriores, além da identificação precoce das crianças com dificuldades relacionadas às HA. Estimular a audição de pré-escolares pode favorecer não só o seu desenvolvimento, mas também pode reduzir os impactos negativos do TPA na comunicação, aprendizagem e habilidades sociais.

Aprender a ler implica a aquisição de habilidades dos processamentos fonológico, ortográfico e semântico. Durante a leitura, o SNC transforma representações gráficas em representações mentais sonoras e as associa aos seus significados^(30,31)^. A transição das crianças entre as séries escolares funciona como uma cascata de acontecimentos que irá, pouco a pouco, prepará-la para essa grande aquisição.

Ao pensarmos na aquisição das habilidades leitoras, precisamos observar, também, as habilidades adquiridas entre os domínios de ambas as formas de comunicação, ou seja, os elementos preditores da leitura. Ao avaliarmos o processamento fonológico (consciência fonológica, acesso lexical e memória operacional fonológica), em escolares no último ano da Educação Infantil, podemos observar essa transição de acontecimentos. Isso justifica o fato de que, dentro do protocolo escolhido para ser utilizado neste estudo, ao longo das avaliações (M1 e M2), foram observadas pontuações nulas, como em habilidades fonêmicas (que são esperadas para o Ensino Fundamental), e uma constante evolução nas habilidades silábicas de ambos os grupos estudados. Esses dados foram confirmados pela MANOVA de medidas repetidas, a qual revelou que, independentemente da intervenção realizada, houve um efeito significativo entre a performance observada nos períodos M1 e M2 em todos os testes das habilidades pré leitoras e de leitura (p<0,01), exceto para a prova de síntese fonêmica, na qual a porcentagem de acertos foi zero, em ambos os grupos, tanto para a avaliação M1 quanto para M2 período em que ainda se encontravam na pré escola.

Ao considerarmos o tipo de estimulação realizada (auditiva ou placebo) e as avaliações M1 e M2, houve interação entre essas variáveis (p<0,001***).

A análise univariada acusou diferenças estatisticamente significantes entre as performances dos grupos GEA e GP nas variáveis: Produção de Rima, Identificação de rima, Produção de Palavra e Memória Operacional. Esses achados vão ao encontro do estudo de Carroll et al.^(32)^, o qual afirma que a consciência fonológica pré-escolar pode ser dividida em uma fase inicial, onde a criança é sensível às semelhanças sonoras implícitas dos vocábulos (vocabulário receptivo) e uma fase posterior, onde a criança se conscientiza de segmentos menores, os fonemas (baseada na aquisição anterior). Ou seja, o desenvolvimento das representações fonológicas segue um processo de refinamento, das características globais para as segmentais, por isso podem ser observados entre os pré-escolares e nas fases iniciais do processo de alfabetização.

Neste sentido, as atividades propostas no Programa elaborado neste estudo, ao estimularem o processamento auditivo, parecem ter contribuído positivamente para uma melhor discriminação dos segmentos silábico das palavras e uso desses segmentos, seja na percepção de tais elementos em posição final (ao identificarem melhor a rima), seja no processo de refletir e evocar uma palavra com um determinado elemento sinalizado (identificação de rima e produção de palavra a partir do fonema dado). É válido reforçar que, ao avaliar a produção de palavra a partir do fonema indicado e não do grafema inicial da palavra, o objetivo foi observar a ativação do processador fonológico (por meio da via auditiva) e não ortográfico, uma vez que as crianças se encontravam em fase pré-escolar.

### Limitações do estudo

Como mencionado previamente, previu-se a avaliação auditiva de todos os escolares anteriormente à aplicação dos testes comportamentais de avaliação do processamento auditivo. Sabe-se que, ao ser realizada a avaliação auditiva, a metodologia asseguraria a adequada sensibilidade auditiva e a integridade do sistema tímpano-ossicular, ainda que os testes aplicados não tenham finalidade diagnóstica.

Entretanto, as coletas de dados foram permeadas pela Pandemia Covid-19 e, infortunadamente, os períodos ideais de coleta aconteceram próximos às fases vermelhas e início do retorno às aulas, onde o trânsito de pessoas entre as instituições era desaconselhado. Uma vez que o *notebook* utilizado já estava em posse da avaliadora e os instrumentos de avaliação audiológica formal deveriam ser retirados e devolvidos à Universidade, cuja sede encontrava-se em outro município, priorizando as condições sanitárias e buscando minimizar os impactos dessa decisão à metodologia da pesquisa (que já havia sofrido grande interferência do cenário de saúde), a fim de manter o estudo em andamento, optou-se por aplicar o mesmo nível de sensação para todos os sujeitos participantes.

Novas pesquisas podem ser realizadas utilizando a metodologia inicial, a fim de averiguar, a partir de avaliações formais, se os resultados pós aplicação do Programa se mantém.

## CONCLUSÃO

O Programa de estimulação das habilidades auditivas em ambiente escolar proposto neste estudo, é composto por atividades lúdicas que devem ser aplicadas diariamente durante 25 semanas do ano letivo escolar, sendo incorporado na grade curricular auxiliando no desenvolvimento das habilidades preconizadas pelo Ministério da Educação.

Após a estimulação auditiva, percebeu-se melhor desempenho em figura-fundo auditiva, resolução temporal, identificação e produção de rima e produção de palavra a partir de fonema dado.
